# Rational Development of Liquid Biopsy Analysis in Renal Cell Carcinoma

**DOI:** 10.3390/cancers13225825

**Published:** 2021-11-20

**Authors:** Kate I. Glennon, Mahafarin Maralani, Narges Abdian, Antoine Paccard, Laura Montermini, Alice Jisoo Nam, Madeleine Arseneault, Alfredo Staffa, Pouria Jandaghi, Brian Meehan, Fadi Brimo, Simon Tanguay, Janusz Rak, Yasser Riazalhosseini

**Affiliations:** 1 McGill Genome Centre, McGill University, 740 Doctor Penfield Avenue, Montreal, QC H3A 0G1, Canada; kate.glennon@mail.mcgill.ca (K.I.G.); mah_afarin@hotmail.com (M.M.); antoine.paccard@mcgill.ca (A.P.); ji.nam@mail.mcgill.ca (A.J.N.); madeleine.arseneault@mcgill.ca (M.A.); astaffa@gmail.com (A.S.); pouria.jandaghi@mail.mcgill.ca (P.J.); 2 Department of Human Genetics, McGill University, 1205 Doctor Penfield Avenue, Montreal, QC H3A 1B1, Canada; 3 The Research Institute of the McGill University Health Centre, Montreal, QC H4A 3J1, Canada; nargesabdian@gmail.com (N.A.); laura-m@sympatico.ca (L.M.); bmeehan82@hotmail.com (B.M.); janusz.rak@mcgill.ca (J.R.); 4 Department of Pathology, McGill University, Montreal, QC H3A 2B4, Canada; fadi.brimo@mcgill.ca; 5 Division of Urology, McGill University, Montreal, QC H4A 3J1, Canada; simon.tanguay@mcgill.ca

**Keywords:** renal cell carcinoma, liquid biopsy, NGS, cfDNA, ctDNA, extracellular vesicles, exosomes

## Abstract

**Simple Summary:**

Among patients affected by renal cell carcinoma (RCC), the most common type of kidney cancer, it remains difficult to identify those who are at high risk for relapse or metastasis. This is in part due to the absence of reliable clinical biomarkers and robust methods to capture them. The aim of our study was to develop an improved assay to capture prognostic genomic biomarkers in circulating tumor DNA (ctDNA) in RCC. For this purpose, we first established a next generation sequencing (NGS) assay, targeting genes that are tailored for RCC and that are largely excluded from commercially available assays. Next, we showed the reliable performance of this assay to detect prognostic gene mutations in tumor DNA isolated from plasma, and from extracellular vesicles. Thus, our study provides a resource to facilitate ctDNA analysis for precision medicine in RCC.

**Abstract:**

Renal cell carcinoma (RCC) is known for its variable clinical behavior and outcome, including heterogeneity in developing relapse or metastasis. Recent data highlighted the potential of somatic mutations as promising biomarkers for risk stratification in RCC. Likewise, the analysis of circulating tumor DNA (ctDNA) for such informative somatic mutations (liquid biopsy) is considered an important advance for precision oncology in RCC, allowing to monitor molecular disease evolution in real time. However, our knowledge about the utility of ctDNA analysis in RCC is limited, in part due to the lack of RCC-appropriate assays for ctDNA analysis. Here, by interrogating different blood compartments in xenograft models, we identified plasma cell-free (cf) DNA and extracellular vesicles (ev) DNA enriched for RCC-associated ctDNA. Additionally, we developed sensitive targeted sequencing and bioinformatics workflows capable of detecting somatic mutations in RCC-relevant genes with allele frequencies ≥ 0.5%. Applying this assay to patient-matched tumor and liquid biopsies, we captured tumor mutations in cf- and ev-DNA fractions isolated from the blood, highlighting the potentials of both fractions for ctDNA analysis. Overall, our study presents an RCC-appropriate sequencing assay and workflow for ctDNA analysis and provides a proof of principle as to the feasibility of detecting tumor-specific mutations in liquid biopsy in RCC patients.

## 1. Introduction

Mutational analysis of plasma circulating tumor DNA (ctDNA) for precision oncology has attracted considerable attention over the past decades [1,2,3]. This approach, often referred to as ‘liquid biopsy’, is of interest due to the fact that it can potentially offer a real time access to diagnostic and actionable mutations regardless of the accessibility and number of lesions present in a patient [2]. Therefore, liquid biopsy analysis is believed to be a powerful resource in the management of patients with cancer [1]. Whereas ctDNA analysis is producing promising results in colorectal and other cancers [1,2,3], there has not been much success with liquid biopsy-based analysis of tumor mutations in renal cell carcinoma (RCC), the most common form of kidney cancers, in spite of the hypervascular nature of these tumors. A plausible reason for this is the absence of RCC-relevant genes in commercially available ctDNA analysis assays, which have been used in the previous studies. For example, two recent large-scale (>200 cases) liquid biopsy studies in RCC [4,5] have deployed assays that do not include commonly mutated genes in RCC, including PBRM1, SETD2, BAP1, and KDM5C, whose mutations are associated with clinical outcomes [6]. Thus, an RCC-appropriate liquid biopsy assay, beyond the commercially available platforms, needs to be developed and optimized to enable the interrogation of RCC-relevant genes. Furthermore, previous studies in other cancers have shown that in addition to soluble plasma, the ctDNA-enriched analytes may include circulating extracellular vesicles (EVs) [7,8,9,10], platelets [11] and leukocytes known to contain tumor DNA [12]. These observations highlight the fact that ctDNA analysis requires robust validation in several technical aspects, which need to be tailored to a particular tumor site due to differences in amenable biofluids, abundance and carriers of genomic sequences released from cancer cells. Such clinical grade information is lacking for RCC.

Among other challenges associated with liquid biopsy analysis in RCC is the low concentration of cell-free DNA (cfDNA) in the blood stream as well as the low proportion of ctDNA present within the cfDNA [13]. Somatic mutations of tumors are often present at very low frequencies (<3%) in cfDNA samples [14] and conventional next-generation sequencing (NGS) approaches are not optimized for the detection of variants with allele frequency below 5% [15]. The implementation of DNA barcoding methods, such as unique molecular identifiers (UMI), coupled with deep-sequencing has improved sensitivity for ctDNA detection [14]. However, the optimization of NGS library preparation and bioinformatics pipelines for ctDNA analysis is a prerequisite of success in this setting. All together, these factors compound the uncertainties about whether liquid biopsy analysis can reflect on status of actionable mutations in RCC tumors.

In this study, we used animal models of RCC to investigate various compartments of blood stream for the enrichment of RCC-associated ctDNA to guide pre-analytical sample preparation for liquid biopsy analysis. Furthermore, we developed and optimized an RCC-specific targeted NGS assay for parallel mutational analyses of tumor tissue-derived DNA and cfDNA to enable a comparison between the status of somatic mutations in tumors as well as in liquid biopsy analytes. Finally, we applied our assay to matched tumor, cfDNA, and evDNA trios from eleven RCC patients to assess the feasibility of liquid biopsy analysis for capturing information of potentially actionable somatic mutations.

## 2. Materials and Methods

### 2.1. Cell Culture

The established renal cell cancer cell line 786-O was purchased from the American Type Culture Collection (ATCC; Rockville, MD, USA), and was cultured according to the ATCC recommendations at 37 °C in humidified air with 5% CO_2_. Cells were transfected with pLenti CMV V5-LUC Blast (w567-1) (addgene #21474, Watertown, MA, USA) using Lipofectamine 3000 (Invitrogen, Waltham, MA, USA) following the manufacturer’s instructions. Stably tagged cells were selected following incubation in medium supplemented with 8 µg/mL blasticidin (Sigma-Aldrich, St. Louis, MI, USA) for 15 days.

### 2.2. Animal Models of RCC

We established orthotopic models of ccRCC by injecting labelled 786-O cells into the subrenal capsule of immune-deficient mice using methods described by Tracz et al. [16]. Briefly, female YFP-SCID mice [17] aged six to eight weeks were anesthetized with isoflurane, and a small incision was made between the last rib and the hip joint of a mouse positioned in right lateral recumbency. After popping up the kidney, an ultra-fine needle was inserted into the lower pole of the kidney and advanced until the needle’s point reached just below the renal subcapsule. One million viable cells mixed with matrigel were slowly injected (volume: 10 μL). After injection, the abdominal wall was closed with a re-absorbable suture and the skin secured with surgical staples. Tumor growth and metastatic disease progression was monitored weekly through luminescence as described previously [18]. The mice were sacrificed after development of metastasis and primary tumors were collected and stored at −80 °C. Blood samples were taken via the inferior vena cava (IVC) using 3.8% sodium citrate as anticoagulant, and were centrifuged to separate plasma and buffy coat samples. For EV preparation blood was centrifuged at 200× *g* for 20 min to sediment blood cells, while the upper portion was transferred to another tube and centrifuged at 1500× *g* for 20 min to remove platelets (platelet-poor plasma) before being passed through a 0.45 µm filter, following by ultracentrifugation as described below. All in vivo experiments were performed according to the Animal Use Protocol (AUP) approved by the Institutional Animal Facility Care Committee and following Guidelines of the Canadian Council of Animal Care (CCAC).

### 2.3. Collection of Blood Samples

Patient blood samples were drawn directly prior to surgery into K2 EDTA (BD, Franklin Lakes, NJ, USA) (cfDNA) and Citrate (BD, Franklin Lakes, NJ, USA) (evDNA) tubes. The tubes were inverted to mix and stored at 4 °C until centrifugation. The blood samples were centrifuged within 60 min of collection at 2000 RCF for 15 min at 4 °C to separate plasma from buffy coat and erythrocyte layers. Plasma and buffy coat fractions were stored in 2 mL cryovials at −80 °C until DNA isolation.

### 2.4. Isolation of EV DNA from Blood Samples

Plasma prepared from mouse or patient blood samples was used for isolation of extracellular vesicles using ultracentrifugation. Briefly, platelet-poor plasma samples (~500 μL) were centrifuged at 110,000× *g* for 70 min at 4 °C. The resulting pellet was washed with PBS and was centrifuged at 110,000× *g* for 70 min at 4 °C for a second time to precipitate EVs. DNA was extracted from EV pellets using the QIAamp DNA Micro kit (Qiagen, Hilden, Germany), following the manufacturer’s instructions.

### 2.5. Digital Droplet PCR (ddPCR)

Digital droplet PCR assays were established as described earlier [12] for the following specific VHL mutation that is present in 786-O cells in consultation with IDT (Integrated DNA Technologies, Coralville, IA, USA): VHL, c.311delG, p.G105fsX55. Mutation-specific primers, gblocks and probes (Table S2) for these mutations were designed and purchased from IDT. DNA samples were subjected to ddPCR for detection of the VHL mutation according to instructions provided by BioRad. Annealing temperature and cycling conditions were optimized, LOD and assay sensitivity were determined using serially diluted gBlocks. Data analysis was performed using QuantaSoft software following the manufacturer’s instructions. 900 nM probes and 250 nM primers were mixed with 2×  Droplet PCR Supermix (Bio-Rad Laboratories, Hercules, CA, USA), 6 ng of template DNA, and H2O to generate 20 μL for each reaction. The reaction mixture was placed into the sample well of an DG8 cartridge (Bio-Rad, Hercules, CA, USA). 70 μL of droplet-generation oil was loaded into the oil well, and droplets were formed in the droplet generator (BioRad). After processing, the droplets were transferred to a 96-well PCR plate (Eppendorf, Hamburg, Germany). The PCR amplification was carried out on C1000 TouchTM Thermal Cycler (Bio-Rad) with the following thermal profile: hold at 95 °C for 10 min, 40 cycles of 95 °C 30 s, 55 °C 1 min (ramp 2 °C/s), and 72 °C 30 s, and 1 cycle at 98 °C for 10 min, and ending at 4 °C. After amplification, the plate was loaded on the droplet reader (Bio-Rad) and the droplets from each well of the plate were read automatically. QuantaSoft software was used to count the PCR-positive (FAM channel) and PCR-negative (HEX channel) droplets to provide absolute quantification of target DNA.

### 2.6. Isolation of Genomic and Soluble Cell-Free DNA

Buffy coat, tumor tissues, and plasma samples for 11 RCC patients were provided by McGill RCC biobank (Table 1). All samples were received following obtaining written consents from the patients and after approval of the study by McGill University Health Centre Research Ethics Board (MUHC REB). Genomic DNA was isolated from buffy coats (control) and frozen tumor tissue using DNeasy Blood and Tissue kit (Qiagen, Hilden, Germany). We used the same kit for isolation of DNA from mouse buffy coat and tumor samples. Soluble cell free DNA was isolated from 4 mL and 500 uL of patient and mouse plasma samples, respectively, using the QIAseq cfDNA All-in-One kit (Qiagen), following manufacturer’s instructions. EV DNA was isolated from plasma sample as described above. Isolated DNA was quantified using Quant-iT PicoGreen dsDNA assay.

### 2.7. Targeted Sequencing

Prior to library preparation, gDNA was sheared by the Covaris ultrasonicator to an average peak size of 350 bp. Genomic DNA libraries were generated using the Lucigen NxSeq AmpFree library preparation kit, with eight PCR cycles added according to the manufacturer’s guidelines for optional PCR amplification. xGen Dual Index UMI Adapters were added during the library preparation. cfDNA and evDNA libraries were generated using the xGen Prism DNA library prep kit (IDT, Coralville, IA, USA), following the manufacturers guidelines and using the included adapters. Libraries were quantified by qPCR, and the average size fragment was determined using a LabChip GX (PerkinElmer, Waltham, MA, USA). Target enrichment was performed using the xGen Hybridization and Wash Kit (IDT, Coralville, IA, USA) using a custom hybridization panel for RCC (IDT). The enriched libraries were sequenced on a NovaSeq 6000 (paired-end 150).

### 2.8. Synthetic cfDNA Library Preparation

Synthetic liquid biopsy samples were generated using the Seraseq ctDNA Mutation Mix (v2, AF2%) spiked into the Seraseq ctDNA Mutation Mix WT at known allele frequencies of 0.1%, 0.5%, and 1%. Libraries were prepared using the Lucigen NxSeq AmpFree, xGen Prism, and QIAseq cfDNA library kits, following the manufacturers guidelines for each kit. A PCR module was added to amplify libraries generated from the Lucigen NxSeq AmpFree kit. Libraries were quantified by qPCR, and the average size fragment was determined using a LabChip GX (PerkinElmer, Waltham, MA, USA). The synthetic libraries generated by the xGen Prism kit were pooled for hybridization capture with a custom hybridization panel using the xGen Hybridization and Wash Kit. The hybridization capture was quantified by qPCR, average size fragment was determined using the LabChip GX, before sequencing on a NovaSeq 6000 (paired-end 150).

### 2.9. Bioinformatic Analysis

Sequencing reads were processed using the GenPipes DNA-Seq High Coverage pipeline [19], with adaptations made for UMI-handling and generation of consensus sequences. Adapters and low-quality reads were removed by Trimmomatic [20], and reads were aligned using bwa-mem2 [21] to the human genome build GRCh37. UMIs were processed using fgbio [22] following the analysis guidelines for xGen Dual Index UMI adapters (IDT) to generate consensus reads. Indel realignment and mate-pair fixing was performed using GATK [23] and Picard [24]. Somatic calls were generated using VarScan2 [25] as well as VarDict [26]. Functional annotation of the somatic calls was added by snpEff [27], and genomic annotation by Gemini [28]. Matched patient normal (buffy coat) samples were used to eliminate germline variants. Non-silent somatic calls underwent manual validation in integrative genomics viewer (IGV) [29] to identify somatic variants present in tumor tissue, circulating cell free DNA, and cell free DNA isolated from extracellular vesicles.

### 2.10. Statistical Analysis

Pearson’s correlations were used to assess relationships between gene-specific proportions of sequencing read in different sample types. Differences in library yields were evaluated using Welch’s *t*-tests.

## 3. Results

Tumor DNA may be present in several biofluid fractions such as liquid phase (e.g., plasma), EVs (including exosomes), and cells (platelets and leukocytes). To establish a liquid biopsy assay appropriate for ctDNA analysis in RCC, we sought to first identify the most informative biofluid compartment for ctDNA analysis in RCC, and then optimize an RCC-appropriate NGS approach for the detection of somatic mutations in tissue and liquid biopsy samples.

### 3.1. Characteristics of the ctDNA Repertoire in RCC Xenografts

**Figure 1:**
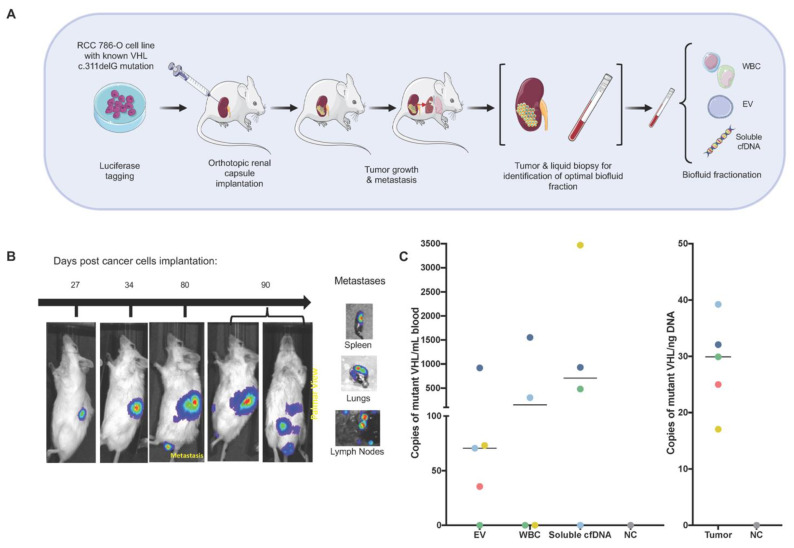
Characterizing the repertoire of ctDNA in RCC. (**A**) Schematic presentation of the in vivo experiments to optimize sample processing for liquid biopsy analysis in RCC. (**B**) Tumor developments in nude mice after orthotropic implantation of 786-O cells. Five mice were implanted, and one animal is shown as an example. Blood samples were drawn after the development of metastatic tumors. (**C**) The presence of the VHL c.311delG mutation in human DNA was interrogated in different blood compartments (left) and in tumor tissues (right) using ddPCR. Each dot represents copy number of mutant allele per mL of blood used for DNA isolation from each animal, or per ng of DNA isolated from tumor tissue. Dot colors represent individual animals. NC: negative control.

In RCC patients ctDNA represents a modest fraction of cfDNA in blood [13]. We questioned whether specific compartments of blood may be enriched for RCC ctDNA, and thereby be more appropriate for liquid biopsy analysis. To minimize technical caveats that originate from the presence of wild-type (background) cfDNA, released by non-cancer cells, we developed orthotopic xenograft models of ccRCC (*n* = 5 animals), which served as a tool for an unambiguous detection of tumor (human) DNA in all fractions of mouse blood (Figure 1A). These models were developed using luminescently-tagged 786-O cancer cells with known RCC-specific mutations, including VHL c.311delG. Following the development of metastatic RCC lesions, blood was collected and subjected to fractionation to isolate blood cells (including leukocytes/WBCs), EVs and soluble cfDNA (Figure 1B). To examine these liquid biopsy fractions for the presence of ctDNA, we used digital droplet PCR (ddPCR) to interrogate them for the aforementioned VHL mutation. This analysis revealed the presence of the mutated DNA in all of the examined blood fractions (Figure 1C). However, while at least 75% of the examined EV samples (four out of five animals) and soluble cfDNA (three out of four animals) fractions were positive for the VHL mutation, we detected this mutation in only 50% (two out of four animals) of tested blood cell fractions. Therefore, we focused on soluble cfDNA and EVs fractions for the analysis of patients’ liquid biopsy material.

### 3.2. Development of the RCC-Appropriate Targeted NGS Assay

**Figure 2:**
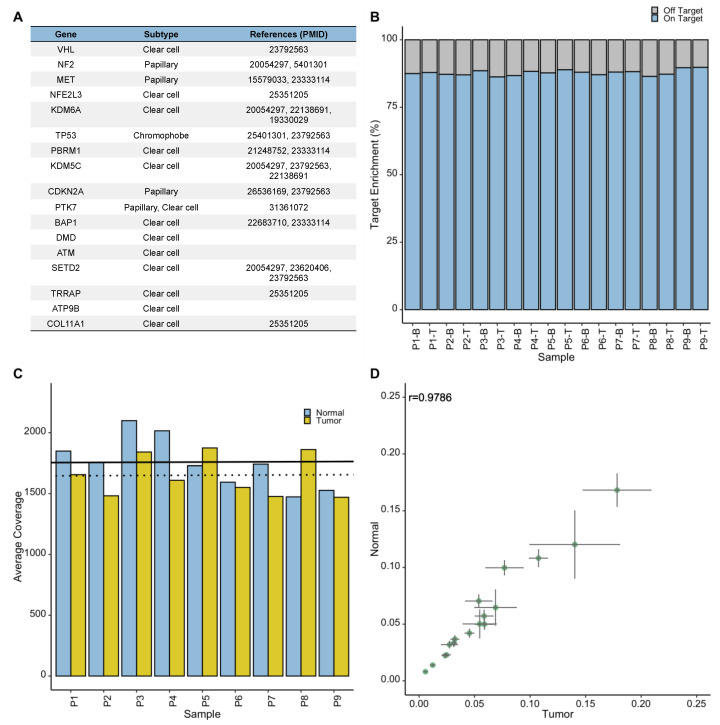
Development and evaluation of the RCC-appropriate NGS assay. (**A**) The RCC assay was designed to target genes that are commonly mutated in RCC tumors, have demonstrated prognostic value in RCC, or have shown association to treatment response in previous large-scale studies. (**B**) The assay showed high efficacy for capturing the targeted regions, with average on-target rates of 87.8% (SD of 0.01) across 18 samples, pooled together prior to the capture. (**C**) Average sequencing coverage is shown for the same 18 samples (9 tumor-normal pairs), included in a given hybridization capture, with average sequencing coverage depths of 1646× and 1753× of the targeted regions for tumor (dotted line) and normal (solid line) DNA samples, respectively. (**D**) High Pearson’s correlations (r > 0.97, *p* < 0.0001) between gene-specific proportions of sequencing reads from tumor and normal samples demonstrates that assay performance is not biased by sample type. Error bars show SD within sample types. SD: standard deviation.

To enable comparison between tumor tissue and liquid biopsies for the status of potentially actionable somatic mutations in RCC, we sought to develop an NGS assay that is compatible with both genomic DNA (gDNA) and cfDNA samples. Therefore, using custom IDT xGen Lockdown Probes, we designed a targeted NGS panel to capture the entire coding regions and exon-intron boundaries of a gene panel, including VHL, PBRM1, SETD2, BAP1, TP53, ATM, KDM5C, DMD, CDKN2A, MET, NF2, KDM6A, NFE2L3, PTK7, TRRAP, ATP9B, and COL11A1. These genes are commonly mutated in RCC tumors (e.g., VHL in ccRCC or MET in papillary RCC), and some of them possess prognostic potential based on the previous large-scale genomic studies [6,30,31] (Figure 2A). Thus, this panel can serve for both diagnostic and prognostic purposes. First, we evaluated enrichment efficacy of the panel for the target genes by generating NGS libraries from high-quality gDNA samples using Lucigen NxSeq AmpFree assay, and subjecting them to the capture panel, followed by sequencing (average depth 1699×). These DNA samples were isolated from buffy-coat (control) or fresh-frozen RCC tumors procured from patients, enrolled in the McGill RCC biobank projects (Table 1). Sequencing results confirmed the average on-target rates of 87.8% across all samples within a capture (Figure 2B), demonstrating the reliable performance of the capture panel to enrich for the desired genes, with no significant difference in sequencing coverage of tumor and normal samples (*p* = 0.255) (Figure 2C). Likewise, matched tumor-normal pairs exhibited high correlations (r > 0.97) for gene-specific proportion of sequencing reads (Figure 2D), indicating that the capture performance is not biased toward sample type and maintains a stable performance across multiple samples. Next, we identified somatic mutations within the gene panel by comparing mutation profiles of tumors to those of their matched blood-driven germline DNA samples (Table S1). Our analysis revealed high prevalence of VHL (82%, 9/11), PBRM1 (73%, 8/11) and SETD2 (36%, 4/11) non-silent mutations in our samples, in line with previous reports [30,31]. These observations demonstrated the capability of the assay to detect somatic mutations in RCC-relevant genes.

### 3.3. Optimization of the NGS Assay for ctDNA Analysis

**Figure 3:**
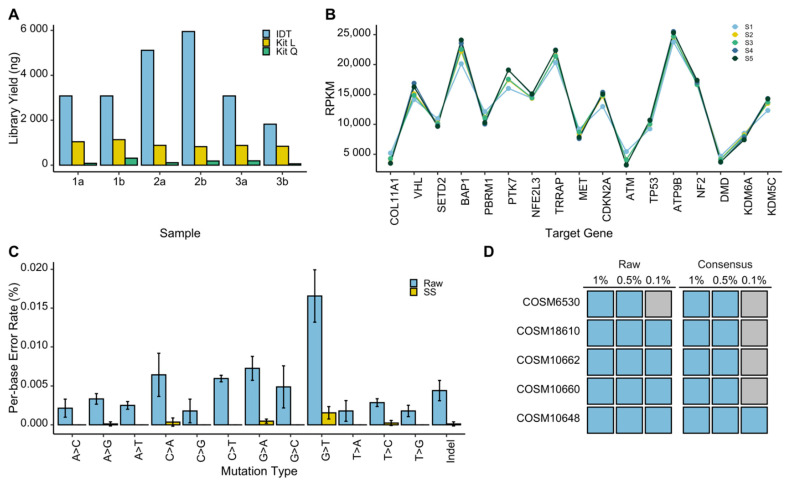
Optimization of the RCC NGS assay for liquid biopsy analysis. (**A**) The comparison between yields of NGS libraries generated by three different methods using 10 ng of the control cfDNA as the starting material is shown. Three individual cfDNA samples tested are identified by numbers (1–3), and technical replicates are marked by letters (a and b). The IDT Prism method resulted in far greater library yields compared to Lucigen NxSeq AmpFree (Kit L) (*p* = 0.007) and Qiagen QIAseq (Kit Q) (*p* = 0.002) methods. (**B**) Reproducible performance of the capture panel was examined by comparing gene-specific number of reads per kilobase per million reads (RPKM) values between five replicates (S1–S5) generated from control cfDNA sample using the IDT Prism method. (**C**) The effect of unique molecular identifiers (UMIs) on reducing sequencing errors is shown. False positive rates were assessed with (Consensus) and without (Raw) using UMIs (*n* = 5). Implementation of UMIs decreased false positive rates across all substitution mutation classes and in indels. (**D**) The assay limit of detection (LOD) was determined by assessing variant drop-out in control samples with known VAFs of 1%, 0.5% and 0.1%. Variant drop-out was observed for allele frequency of 0.1% in both raw and consensus reads. Blue and grey indicate the presence and absence of a given true mutation, respectively.

Next, we sought to optimize the workflow of our RCC-specific targeted assay for the reliable analysis of cfDNA. Given the low abundance of ctDNA in the limited amount of cfDNA, which can be isolated from plasma of RCC patients, we focused our efforts on two aspects: (1) identifying an effective library preparation approach for cfDNA analysis, and (2) improving detection sensitivity by ultra-deep sequencing coupled with the implementation of unique molecular identifiers (UMIs) in the library preparation workflow to correct for sequencing errors. To this end, we compared the efficacy of the Lucigen NxSeq AmpFree, Qiagen QIAseq cfDNA, and IDT PRISM methods for generating NGS libraries from synthetic cfDNA control samples, which are commercially available. Furthermore, to assess the sensitivity for mutation detection we extended our analysis by including control cfDNA templates with known variant allele frequencies (VAFs) (0.1%, 0.5%, and 1%) for five cancer-associated TP53 mutations (see the ‘Methods’ section for details). Among the examined library preparation methods, the IDT PRISM resulted in the greatest library yield in all replicate samples (Figure 3A). In addition, the analysis of library profiles confirmed the high quality of the libraries generated by the IDT PRISM approach. Therefore, we subjected these libraries to our targeted capture panel, followed by deep-sequencing (>70 million reads per sample) in order to assess the capture efficacy and stability across multiple samples. This was assessed by evaluating the number of sequencing reads attributed to each target gene, across five replicate samples. While the reads per kilobase of target region, per million mapped reads (RPKM) values showed variable sequencing depths for individual target genes, it maintained a consistent trend across all replicates (Figure 3B). This confirmed the ability of the enrichment panel to capture all RCC-genes from control cfDNA samples, and the stability of assay performance.

To optimize our bioinformatics pipeline for mutation detection and establishing the sensitivity of our liquid biopsy assay, we first examined the utility of the UMIs for reducing sequencing errors. For this, we focused on the TP53 gene, for which we knew the exact location and type of five somatic mutations in the synthetic cfDNA controls, and therefore were able to distinguish them from sequencing errors. Per-base error rates were generated across TP53 for each substitution mutation class and total mutations including indels from sequencing data, processed once without the implementation of UMIs (raw), and another time with UMIs to generate error-free consensus sequences (SS). Comparisons between the raw and consensus sequences revealed a substantial reduction in per-base error rates in all substitutions classes as well as in indels (Figure 3C).

Although the implementation of UMIs vastly decreases the rate of false positives, it can also cause variant drop-out at very low allele frequencies due to the greater stringency. To determine the limit of detection (LOD) for our assay, we investigated variant dropout of the known TP53 mutations in synthetic cfDNA samples with known VAFs for these mutations ranging from 0.1–1.0%. By obtaining 70 million reads per sample, we were able to detect the TP53 mutations with VAFs of 0.5 and 1%, whereas we observed dropout of the same mutations at VAF of 0.1%. (Figure 3D). Therefore, at VAFs ≥ 0.5% we were able to detect all true TP53 variants in the synthetic cfDNA controls and minimize the number of false positives by implementing UMIs.

### 3.4. Assay Performance in RCC Liquid Biopsies

**Figure 4:**
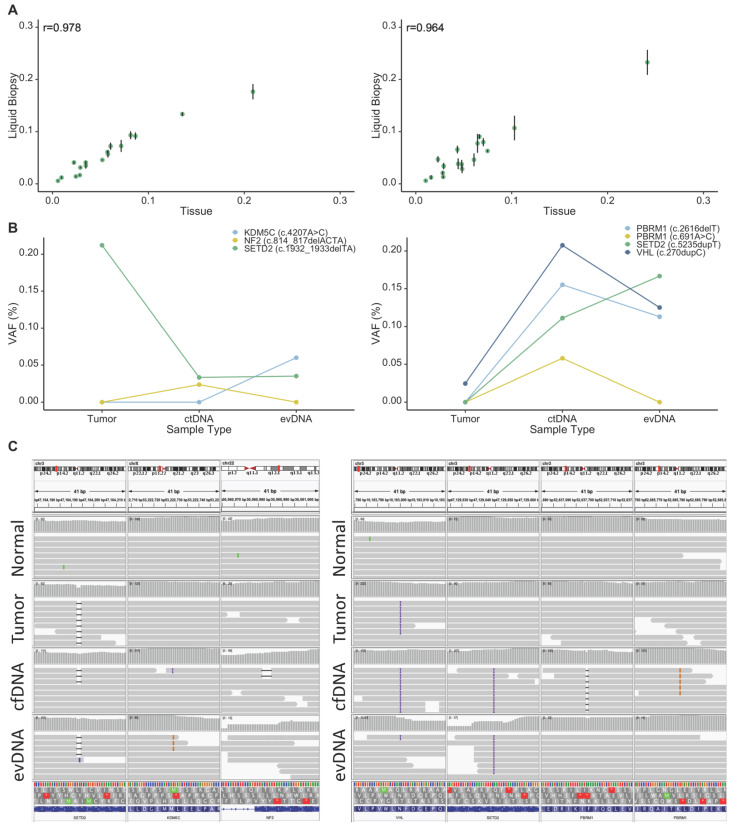
Evaluating the feasibility of detecting tumor somatic mutations in liquid biopsy in RCC. (**A**) In-patient comparisons between tumor and liquid biopsy-driven DNA samples showed high Pearson’s correlation in capture distribution. Example data is shown for two patients (r = 0.978, *p* < 0.0001 and r = 0.964, *p* < 0.0001, for P10 and P11 respectively). Error bars show SD within liquid biopsies. (**B**) Somatic variants identified in both tumor tissue, and in liquid biopsy fractions for patients P10 (left) and P11 (right) at variable allele frequencies. The SETD2 variant in patient P10 was detected at low frequencies in both ctDNA and evDNA, whereas the KDM5C variant was detected only in the evDNA fraction, and the NF2 variant was detected only in the cfDNA fraction. The VHL variant identified in P11 tumor, showed an increased allele frequency in liquid biopsy fractions, whereas mutations in PBRM1 and SETD2 were only detected in liquid biopsy fractions. (**C**) Somatic variants identified in tumor tissue, ctDNA, and evDNA visualized using Integrative Genomics Viewer (IGV) for both patient P10 (left) and P11 (right). These somatic variants were not present in the normal (buffy coat) samples (top panel). SD: standard deviation.

Following assay development and optimization using synthetic cfDNA controls, we extended our study to examine the performance of the assay in capturing somatic mutations in liquid biopsy samples from our patient cohort of eleven RCC patients (Table 1). The number of patients was limited so as to achieve assay validation (present study) before a larger clinical cohort could be rigorously powered and examined. Therefore, we sequenced captured targets in cfDNA isolated from plasma and circulating EVs (aiming at 100 M reads/per sample, resulted in more than 5000× depth of on-target coverage) from each patient in order to enable a comparison between liquid biopsy fractions as well as between them and the tumor. For this purpose, we first compared capture efficacy between gDNA and cfDNA fractions for each patient by analyzing proportions of sequencing reads that mapped to each gene. We observed high correlations (r > 0.95) between liquid biopsy and tumor DNA samples for gene-specific proportion of sequencing reads (examples are shown in Figure 4A), confirming that the performance of the gene-enrichment assay is not dependent on the sample type, and that the assay can be used to compare genetic data between tumor and liquid biopsy samples. Next, we detected somatic mutations in liquid biopsy DNA samples by comparing them to germline DNA isolated from buffy coat samples.

Given the different presentation patterns of ctDNA between patients with advanced tumors and those affected with early-stage cancers [13,32,33], we investigated our results in these groups separately. Amongst the 11 patients included in our study, 4 were affected with advanced tumors (stages T3 and T4; P3, P8, P10 and P11). We detected at least one tumor-specific somatic mutation in liquid biopsy fractions from all of these patients (100%, 4/4; Table 1 and Table S1). In patient P3 (stage T3a), we detected a frameshift variant in SETD2 (c.913dupA) in both soluble cfDNA and evDNA. Similarly, in patient P8 (stage T3b) we detected two somatic missense mutations in COL11A1 and BAP1 both liquid biopsy fractions. Interestingly in liquid biopsy samples from P10 and P11, who are affected with T4 stage tumors, we captured all tumor-specific somatic mutations in both cfDNA and evDNA fractions (Table 1 and Table S1, Figure 4B). An interesting observation was about a frameshift mutation, c.270dupC, in VHL in patient P11, where allele frequency of this mutation was much higher in both liquid biopsy fractions compared to that of tumor DNA (3%, 20%, and 21.5% in tumor, cfDNA, and evDNA, respectively (Figure 4B).

Furthermore, we observed appearance of novel mutations, which were not present in the tumor tissues of patients P10 and P11 in their liquid biopsies. For P10, these were a missense mutation, c.4207A > C, in KDM5C in the evDNA, and a frameshift mutation in NF2, c.814_817delACTA in the cfDNA that were not captured in tumor sequencing data. Likewise, we observed frameshift mutations in PBRM1 (c.2616delT) and SETD2 (c.5235dupT), and a missense mutation in PBRM1 (c.691A > C) in liquid biopsy fractions of P11, that were not present in the sequencing data of the primary tumor (Figure 4C).

In patients with low stage RCC (stages T1–T2), most somatic mutations captured in the tumor tissue were not detectable in either cfDNA or evDNA; however, we did detect a somatic stop-gain mutation of VHL (c.481C > T) in cfDNA sample of patient P2 who was affected by stage T1a tumor. These results suggest that optimized liquid biopsy protocol is suitable for interrogating RCC progression in patients with high stage cancer. However, even in a limited number of cases examined the differential abilities of liquid biopsy analytes to carry mutant signatures (cfDNA, evDNA) are readily observed and this factor should be considered in designing future clinical studies.

## 4. Discussion

The utility of ctDNA analysis in the management of kidney cancers has not yet been deeply explored, in part due to the lack of appropriate platforms that enable side-by-side interrogation of somatic mutations in RCC-relevant genes in tumors and in liquid biopsies. In this study, we developed an RCC-focused NGS assay, and optimized it for parallel tissue and liquid biopsy analyses of RCC-relevant mutations. It has been suggested that the most promising use of ctDNA analysis in RCC is as a surveillance biomarker for metastases and to determine the risk of disease recurrence [34]. Accordingly, the ability to capture the mutational status of RCC-relevant genes, including VHL, BAP1 and PBRM1, is critical for the clinical utility of liquid biopsy analysis in RCC, as the presence of somatic mutations in these particular genes alone or in combination with each other are indicative of distinct disease outcomes [35].

In addition, we provided proof-of-principle evidence on the feasibility of capturing tumor-specific diagnostic and prognostic genomic biomarkers in blood-based liquid biopsies in RCC using our assay. Therefore, our assay provides a reliable platform to address key questions that should be investigated in order to establish robust liquid biopsy strategies for RCC. One of such questions is the interpretation of discordance between tumor DNA and ctDNA analysis results. The discordance between somatic alterations detected in RCC tumor tissues and those detected in ctDNA has been suggested to stem from RCC clonal and spatial heterogeneity, long time intervals between tissue and liquid biopsy sampling, or simply low sensitivity of NGS approaches used for ctDNA analysis [34]. The latter is particularly plausible when a somatic mutation is present in tumor DNA but not detected in the liquid biopsy. An explanation for this is that somatic mutations can be present at extremely low allele frequencies in liquid biopsies for different reasons, including the low fraction of ctDNA within cfDNA [14]. Indeed, a recent study has shown that the abundance of ctDNA in RCC is very low, as compared to other cancers [32] and often ctDNA is detectable in less than 50% of RCC patients [13,33]. These findings highlight the need for the amplification of starting cfDNA material and applying ultra-deep sequencing of generated NGS libraries, both also known to induce errors in sequencing results [36]. It is reasonable to suggest that poor ctDNA detection is due to methodological factors as there are no compelling biological reasons why highly vascularized RCC lesions would not release DNA sequences into blood, passively or actively and in various forms.

Therefore, while exploiting strong amplification and ultra-deep sequencing, we thoroughly investigated the associated technical errors, and corrected for them by implementing molecular tagging through the use of UMIs in library preparation and bioinformatics workflows. Furthermore, the patients included in our study had blood drawn directly prior to surgery, ensuring that the liquid biopsy and tissue sampling are representative of the same time point in tumor evolution. We optimized sample workflow to establish comparable performances in patient matched tumor-normal and liquid biopsy samples, ensuring that potential differences in mutational profiles between sample types are not due to shortcomings in experimental procedures, and rather are reflecting true differences between these sample types. As such, we showed that the assay generates comparable results when applied to tumor DNA and cfDNA from RCC patients with advanced disease (stages T3 or T4 tumor). Notably, at least one somatic variant identified in the primary tumors of patients with advanced RCC was also captured by our assay in the liquid biopsy fractions. Although very preliminary, due to the limited number of examined samples thus far, this result is promising, as it indicates that our assay does not suffer from major caveats that may result in false-negative observations in liquid biopsy analysis.

Strikingly, we also captured somatic mutations in liquid biopsy that were not present in the tumor DNA. The observation that these mutations were present in both patient-matched ctDNA and evDNA fractions of liquid biopsy argues against the possibility that these are false-positive calls. We believe that these mutations are true somatic mutations that were not captured by tumor DNA analysis. In fact, it has been suggested that cfDNA is a better representation of the primary tumor heterogeneity [33,37,38], as circulating tumor DNA sequences are believed to be shed from the entire tumor, while DNA isolated from tumor cells may be spatially biased by the sampling process and limited by availability of tissue material. This is of paramount importance when considering the clinical utility of somatic mutational analysis, given the spatial heterogeneity that is a hallmark of RCC tumors [35,39]. Indeed, ctDNA was shown to be a better predictor of drug resistance than tumor tissue in other cancer contexts [38]. Moreover, liquid biopsy offers an opportunity to collect multiple longitudinal samples in real time and in a non-invasive manner. As such, liquid-biopsy approaches, as compared to direct tumor biopsy, may possess considerable advantages in developing genomic-based precision medicine in RCC. However, this possibility needs further investigation through parallel analyses of patient-matched tumor DNA and cfDNA samples in large sample sets and using assays that generate comparable results from these sample types, such as the method that we presented in this study. There is also a need to further optimize the detection of ctDNA in patients with low stage RCCs. Previous studies have shown that tumor-fraction in plasma can be enhanced by size-selection of DNA fragments, thus increasing the sensitivity to detect somatic mutations in renal cancer and other cancers with low amounts of ctDNA [40]. Future studies are warranted to examine whether this approach can improve the detection of ctDNA in RCC patients with early-stage tumors.

By emphasizing the assay performance and sensitivity, we explored solutions for some of the major caveats of applying liquid biopsy to renal cancer. Notably, and of equal importance, we also showed that clinically informative somatic mutations in RCC may be present not only in form of soluble ctDNA but also be encapsulated in EVs, suggesting that the analysis of both fractions may provide complementary or confirmatory results for liquid biopsy analysis in RCC. Indeed, previous studies have shown that in some cancers, tumor-derived extracellular vesicles are enriched in tumor DNA [41]. EVs serve as carriers of important clinical information, including driver mutations, drug resistance markers, and determinants of immunoregulation [42]. Additionally, they may have advantages for biomarker analysis as they protect their cargo from degradation [42]; however, harnessing information from EVs for liquid biopsy requires sensitive assays given the low abundance of circulating cancer-related EVs. Our results from RCC animal studies, as well as those on patient material supported the evidence from previous studies [4,5,32] that soluble ctDNA is appropriate for interrogating prognostic biomarkers in RCC; however, also indicated that evDNA is a strong candidate and should be considered in future investigations and with room for refinements (multiplexing, selective capture, others). Taken together, the current study provides a robust workflow and rationale for larger future studies to investigate the utility of ctDNA and evDNA for capturing diagnostic and prognostic biomarkers in RCC.

## 5. Conclusions

In this study, we developed and optimized an RCC-appropriate NGS assay applicable to both tumor tissue and liquid biopsy fractions. The assay showed consistent performance in all sample types originating from the same patient (buffy coat, tumor tissue, cfDNA, and evDNA), as well as consistent performance within each sample type. We successfully applied the assay to matched samples from RCC patients with variable clinical features, and captured relevant somatic variants present in primary tumors in both ctDNA and evDNA of patients with advanced tumors. Notably, we demonstrated that ctDNA encapsulated in EVs may contain clinically-relevant mutations in RCC. Furthermore, our assay is the first NGS assay tailored specifically to renal cell carcinoma, including a panel of genes with both diagnostic and prognostic values. This study serves as a demonstration of the capabilities of ctDNA in capturing relevant biomarkers, and lays groundwork for larger studies to further refine the utility of liquid biopsy for enhancing personalized care in RCC.

## Figures and Tables

**Table 1:** Information about 11 enrolled patients in this study. Clinical features of tumors as well as genes affected by somatic mutations in each tumor are provided (see Table S1 for details of somatic mutations).

Patient	Sex	Age	RCC Subtype	Pathological Tumor Stage	Pathological Tumor Grade	Mutated Genes
P1	Male	63	ccRCC	T1b	4/4	VHL, PBRM1
P2	Male	57	ccRCC	T1a	3/4	**VHL**, PBRM1
P3	Female	62	ccRCC	T3a	3/4	VHL, **SETD2**, PBRM1, MET
P4	Female	78	ccRCC	T1a	3/4	VHL, PBRM1
P5	Female	58	ccRCC	T1a	2/4	VHL, COL11A1, SETD2, PBRM1, TRRAP, ATM
P6	Male	66	ccRCC	T1a	3/4	VHL, PBRM1, KDM5C
P7	Female	58	ccRCC	T1a	2/4	VHL
P8	Male	77	ccRCC	T3b	4/4	**COL11A1**, **BAP1**, PBRM1
P9	Female	61	ccRCC	T1a	1/4	VHL
P10	Female	57	Unclassified RCC	T4	4/4	**KDM5C** *, **SETD2**, **NF2** *
P11	Male	43	ccRCC	T4	4/4	**VHL**, **PBRM1** *, **SETD2** *

Note: * only detected in liquid biopsies. Genes whose tumor-specific somatic mutations were detected in liquid biopsies are indicated in bold.

## Data Availability

Details about somatic mutations identified in tumors or liquid biopsy samples are available in Table S1. Raw NGS data is openly available in the European Genome-Phenome Archive (EGA) (Accession code can be obtained from the authors).

## Supplementary Materials

The following are available online at https://www.mdpi.com/article/10.3390/cancers13225825/s1, Table S1: Detailed somatic variant information, including variant annotation and predicted impact for patients P1–P11. Table S2: Mutation-specific probes and primers designed for ddPCR analysis.
